# Mortality of Pandrug-Resistant *Klebsiella pneumoniae* Bloodstream Infections in Critically Ill Patients: A Retrospective Cohort of 115 Episodes

**DOI:** 10.3390/antibiotics10010076

**Published:** 2021-01-15

**Authors:** Matthaios Papadimitriou-Olivgeris, Christina Bartzavali, Alexandra Georgakopoulou, Fevronia Kolonitsiou, Chrisavgi Papamichail, Iris Spiliopoulou, Myrto Christofidou, Fotini Fligou, Markos Marangos

**Affiliations:** 1 Division of Infectious Diseases, School of Medicine, University of Patras, 26504 Patras, Greece; mmarangos@yahoo.com; 2 Department of Microbiology, School of Medicine, University of Patras, 26504 Patras, Greece; chrisbartzavali@gmail.com (C.B.); kolonits@upatras.gr (F.K.); spiliopl@upatras.gr (I.S.); christof@upatras.gr (M.C.); 3 Anesthesiology and Critical Care Medicine, School of Medicine, University of Patras, 26504 Patras, Greece; georgakopouloualexandra@hotmail.com (A.G.); chrisaugi@hotmail.com (C.P.); fflig@yahoo.com (F.F.)

**Keywords:** intensive care unit (ICU), bacteraemia, carbapenemase, carbapenem-resistance, pandrug-resistance

## Abstract

Background: The increased frequency of bacteraemias caused by pandrug-resistant *Klebsiella* *pneumoniae* (PDR-Kp) has significant implications. The aim of the present study was to identify predictors associated with mortality of PDR-Kp bacteraemias. Methods: Patients with monomicrobial bacteraemia due to PDR-Kp were included. *K. pneumoniae* was considered PDR if it showed resistance to all available groups of antibiotics. Primary outcome was 30-day mortality. Minimum inhibitory concentrations (MICs) of meropenem, tigecycline, fosfomycin, and ceftazidime/avibactam were determined by Etest, whereas for colistin, the broth microdilution method was applied. *bla*_KPC_, *bla*_VIM_, *bla*_NDM_, and *bla*_OXA_ genes were detected by PCR. Results: Among 115 PDR-Kp bacteraemias, the majority of infections were primary bacteraemias (53; 46.1%), followed by catheter-related (35; 30.4%). All isolates were resistant to tested antimicrobials. *bla*_KPC_ was the most prevalent carbapenemase gene (98 isolates; 85.2%). Thirty-day mortality was 39.1%; among 51 patients with septic shock, 30-day mortality was 54.9%. Multivariate analysis identified the development of septic shock, Charlson comorbidity index, and bacteraemia other than primary or catheter-related as independent predictors of mortality, while a combination of at least three antimicrobials was identified as an independent predictor of survival. Conclusions: Mortality of PDR-Kp bloodstream infections was high. Administration of at least three antimicrobials might be beneficial for infections in critically ill patients caused by such pathogens.

## 1. Introduction

*Klebsiella pneumoniae* is a Gram-negative, encapsulated, bacterium that colonizes the gastrointestinal tract. Due to its ability to evade the immune system, it can gain entry to other tissues and cause severe infections, mainly urinary tract infections, lower respiratory-tract infection, liver abscesses, and bacteraemias [1]. *K. pneumoniae* has the ability to acquire resistance to different antimicrobials, rendering infections by such multidrug-resistant strains difficult to treat [2].

In the last two decades, carbapenemase-producing bacteria and especially *K. pneumoniae* became prevalent in many regions of the world [3]. The rise of carbapenemase-producing *K. pneumoniae* led to increased use of antimicrobials such as colistin, tigecycline, and aminoglycosides, which subsequently resulted in the emergence of resistance to the aforementioned antimicrobials [4,5]. This led to the isolation of strains resistant to all available antimicrobials, known as pandrug-resistant bacteria (PDR) [6].

While isolation of PDR strains remained rare, nowadays they are prevalent worldwide [6]. They are mainly associated with critically ill patients previously exposed to many antimicrobials [6,7,8,9,10]. Mortality of infections caused by such bacteria is high, since no effective option is available [6,7,8,9,10]. While treatment was based mainly on a combination of different antimicrobials, usually including colistin, no robust evidence exists supporting the superiority of a therapeutic regimen over another [6,7,8,9,10].

The aim of the present study was to describe the clinical characteristics, therapeutic management, and clinical outcome of bloodstream-infections (BSIs) due to PDR *K. pneumoniae* (PDR-Kp) in patients hospitalized in a Greek intensive care unit (ICU).

## 2. Results

Among 412 monomicrobial BSIs due to *K. pneumoniae*, 115 (27.9%) were due to PDR isolates (Supplementary Figure). The majority of infections were primary BSIs (53; 46.1%), followed by catheter-related (35; 30.4%), abdominal infections (11; 9.6%), ventilator-associated pneumonias (8; 7.0%), meningitis (four; 3.5%), urinary tract infections (three; 2.6%), and deep surgical site infections (one; 0.9%).

All 115 isolates recovered from blood cultures were resistant to penicillins, cephalosporins, carbapenems, monobactams, quinolones, sulfamethoxazole-trimethoprim, aminoglycosides, colistin, and tigecycline. Fosfomycin and ceftazidime/avibactam were tested among eight isolates and six isolates, respectively, all of which were resistant. *bla*_KPC_ was the most prevalent carbapenemase gene (98 isolates; 85.2%), followed by co-carriage of *bla*_KPC_ and *bla*_VIM_ (seven; 6.1%), *bla*_VIM_ (six; 5.2%), and *bla*_NDM_ (four; 3.1%).

Thirty-day mortality was 39.1% (45 out of 115 episodes); among the 51 patients that developed septic shock, 30-day mortality was 54.9% (28 patients). The univariate analyses of predictors of 30-day mortality among all patients and those that developed septic shock are shown in Table 1. Multivariate analyses are shown in Table 2. Among all patients, multivariate analysis identified development of septic shock, Charlson comorbidity index, and BSI other than primary or catheter-related as independent predictors of mortality, while a combination of at least three antimicrobials was identified as an independent predictor of survival. Among patients with septic shock, BSI other than primary or catheter-related was identified as an independent predictor of 30-day mortality, while a combination of at least three antimicrobials was identified as an independent predictor of survival.

**Figure 1:**
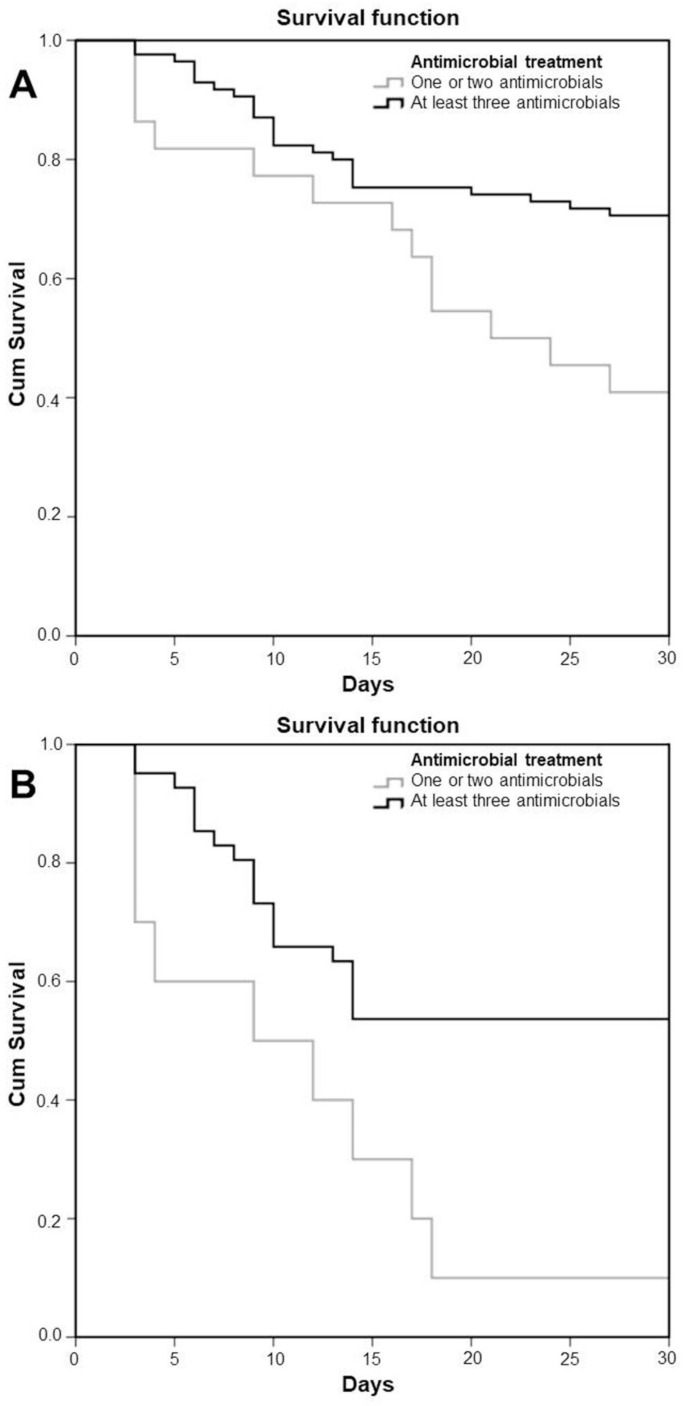
Kaplan–Meier curve of survival probability of patients with PDR-Kp BSIs according to number of administrated antimicrobials. (**A**): all patients (*p* = 0.001); (**B**): patients with septic shock (*p* = 0.023).

Table 3 shows the different antimicrobial regimens and the associated mortality. Figure 1 shows the survival curves of patients with PDR-Kp bacteraemias (Figure 1A) and the subgroup of patients with septic shock (Figure 1B) as a function of the number of administered antimicrobials. A combination of at least three antimicrobials was advantageous over one or two antimicrobials in the whole cohort (*p*=0.001) and in the subgroup of septic shock (*p* = 0.023).

## 3. Discussion

Resistance of carbapenemase-producing bacteria to the remaining antimicrobials is a major emerging healthcare problem. To the best of our knowledge, this is the largest series of PDR-Kp infections. During the ten-year study period (2010–2019), PDR-Kp represented 27.9% of all *K. pneumoniae* bacteraemias (Supplementary Figure). This high percentage of PDR isolates could be attributed to the antibiotic selective pressure in an area with endemic carbapenemase-producing *K. pneumoniae* infections [5], lack of newer antimicrobials, and the revision by the European Committee on Antimicrobial Susceptibility Testing (EUCAST) of tigecycline’s breakpoints rendering most isolates that previously were considered susceptible or intermediate as being resistant to tigecycline [11]. The mortality in our cohort was high (39.1%), especially in patients with septic shock (54.9%), and it was higher than previously reported [6,7,8,9,10]. Most studies were based in cohorts with a limited number of patients infected by different pathogens. Furthermore, in our cohort, all episodes were monomicrobial, which was not the case in previous ones [6,7,8,9,10]. Even though mortality was high in our study, it was significantly lower than the mortality expected when no appropriate antimicrobial treatment is available, most likely because of the synergistic in vivo effect of antimicrobial combination. As previously reported, colistin exhibited synergy when used in combination with other antimicrobials, even against colistin-resistant isolates [12]. A double carbapenem regimen was also associated with better outcomes when used against isolates with high-level carbapenem resistance [13]. Another plausible explanation was that antibiotic resistance comes at a cost of biological fitness. As shown in a previous study, *K. pneumoniae* carbapenemase (KPC)-producing *K. pneumoniae* was practically avirulent as compared to non-carbapenemase-producing *K. pneumoniae* [14]. Choi et al. showed that when hypermucoviscous *K. pneumoniae* acquired colistin resistance, it led to a reduction in capsular polysaccharides production and a significant fitness cost [15].

As previously reported, septic shock was associated with the worst outcomes, especially in infections for which no effective in vitro antimicrobial is available [16,17]. Patients with catheter-related BSIs as compared to other sources (meningitis, intra-abdominal, ventilator-associated pneumonia) were associated with better outcome, underlining the importance of early and appropriate source control in the management of such infections [18,19].

At the present time, no therapy is proven to be superior for patients with PDR-Kp infections [6,7,8,9,10]. Different combinations of antimicrobials have been previously used against PDR-Kp infections with moderate success [6]. Colistin was often used because of in vitro and in vivo data supporting its use in infections caused by PDR pathogens [6,7,8,9,10,12]. In our study, even though the use of at least three antimicrobials was associated with better outcomes, no specific antimicrobial regimen showed a significant superiority (Table 3).

Newer antimicrobials might offer an important adjunction in our already limited armamentarium, since they have shown high rates of in vitro susceptibility in isolates resistant to many currently used antimicrobials. The majority of newer antimicrobials are beta-lactam/beta-lactamase inhibitor combinations with variable activity against different types of carbapenemase genes, a novel cephalosporin (cefiderocol), a novel aminoglycoside (plazomicin), and a novel tetracycline (eravacycline) [20]. Two studies from Greece showed that 100% and 94.3% of *K. pneumoniae* isolates were susceptible to cefiderocol and plazomicin, respectively [21,22]. Such antimicrobials should not be considered as panacea, and their use should be prudent, since excessive use could lead to a rapid change of epidemiology with the replacement of susceptible pathogens by resistant-ones, as previously reported in our ICU after the introduction of ceftazidime/avibactam [23].

This study has several limitations. First, it is a retrospective study conducted in only one center. Even though a moderate number of patients were included, the number of BSIs was significantly higher to previous cohorts [7]. Since no guidelines exist for the treatment of such infections, the administered regimen was at the discretion of the ICU physicians and the infectious diseases consultants, explaining the different approaches seen in the present study. The study was conducted in critically ill patients, so these results may not be directly extrapolated to patients hospitalized in other wards with less severe infections. No time-kill assays were performed to confirm in vitro synergism of different antimicrobial agents.

## 4. Materials and Methods

This retrospective study was conducted during a ten-year period (2010–2019) in the intensive care unit (ICU) of the University General Hospital of Patras (UGHP), Greece. The study was approved by the Ethical Committee of the UGHP (No 858).

Patients with a monomicrobial bacteraemia due to PDR-Kp were included in the study. *K. pneumoniae* was considered PDR if it was resistant to all classes of antibiotics available [24]; isolates from 2010 to 2015 were considered PDR if they were resistant to penicillins, cephalosporins, carbapenems, monobactams, quinolones, sulfamethoxazole-trimethoprim, aminoglycosides, colistin ,and tigecycline; from 2016 to 2017, isolates were considered PDR if they were resistant to the aforementioned antimicrobials plus fosfomycin, whenever the latter was tested; from 2018 to 2019, isolates were considered PDR if they were resistant to the aforementioned antibiotics plus ceftazidime/avibactam. According to the new EUCAST definitions, *K. pneumoniae* strains with minimum inhibitory concentrations (MICs) in the range of “I” were considered susceptible, and such isolates were excluded from our cohort [25]. Multiple episodes of bacteraemia from the same patient were included if a duration of at least two months occurred between two episodes.

Primary outcome was 30-day mortality. Epidemiological data, comorbidities, antimicrobial administration, type of infection, septic shock, and mortality prediction scores (Simplified Acute Physiology Score II (SAPS II), Sequential Organ Failure Assessment (SOFA)) were obtained from patients’ chart reviews and the ICU computerized database (Criticus^TM^, University of Patras, Greece) [17,26]. Primary or secondary BSI was determined in accordance to the Centers for Disease Control and Prevention (CDC) definition [27]. Infection was categorized as sepsis or septic shock according to the new sepsis definition [28]. The date of collection of the first positive blood culture was defined as infection onset.

*K. pneumoniae* isolates from clinical specimens from patients hospitalized in the ICU were identified by the Vitek 2 Advanced Expert System (bioMérieux, Marcy l’ Etoile, France). Antimicrobial susceptibility testing was performed by the agar disk diffusion method against imipenem, meropenem, aztreonam, amikacin, gentamicin, sulfamethoxazole-trimethoprim, and ciprofloxacin. Minimum inhibitory concentrations (MICs) of imipenem, meropenem, tigecycline, fosfomycin, and ceftazidime/avibactam were determined by Etest (bioMérieux), whereas the MIC of colistin was determined by the broth microdilution method. EUCAST criteria were used to interpret susceptibility results [25]. Carbapenemase gene presence (*bla*_VIM_, *bla*_IMP_, *bla*_KPC_, *bla*_NDM_, and *bla*_OXA_) was confirmed by PCR [29,30].

Data analysis was performed with SPSS version 23.0 (SPSS, Chicago, IL, USA). Fisher exact test or the chi-squared test was used for categorical variables and Mann–Whitney *U*-test for continuous ones. Multiple logistic regression analysis was used to identify independent predictors of 30-day mortality in all patients and in the subgroup of patients with septic shock; variables with a univariate *p* ≤ 0.1 were included. In this study, a *p* value < 0.05 was considered significant. Survival curves were obtained using the Kaplan–Meier method.

## 5. Conclusions

The incidence of infections by PDR-Kp was high in an area where carbapenemase-producing *K. pneumoniae* is endemic. Mortality of PDR-Kp BSI was high, especially among patients with septic shock. Administration of at least three antimicrobials might be beneficial for the treatment of BSI caused by such pathogens in critically ill patients, but further studies are needed to elucidate the best therapeutic approach.

## Figures and Tables

**Table 1:** Univariate analyses of predictors of 30-day mortality in patients with pandrug-resistant *Klebsiella pneumoniae* (PDR-Kp) bloodstream-infection (BSI) during intensive care unit (ICU) stay.

Characteristics	All Patients			Patients with Septic Shock		
	Survivors (*n* = 70)	Non-Survivors (*n* = 45)	*p*	Survivors (*n* = 23)	Non-Survivors (*n* = 28)	*p*
Demographics						
Age (years)	51.5 ± 18.6	62.1 ± 15.4	0.003	59.3 ± 18.5	59.4 ± 15.9	0.947
Male gender	49 (70.0%)	26 (57.8%)	0.229	17 (73.9%)	18 (64.3%)	0.552
Comorbidities						
Diabetes mellitus	9 (12.9%)	10 (22.2%)	0.207	2 (8.7%)	6 (21.4%)	0.269
Chronic obstructive pulmonary disease	3 (4.3%)	7 (15.6%)	0.047	0 (0.0%)	4 (14.3%)	0.117
Chronic heart failure	3 (4.3%)	8 (17.8%)	0.023	0 (0.0%)	4 (14.3%)	0.117
Chronic kidney disease	1 (1.2%)	1 (2.2%)	1.000	1 (4.3%)	0 (0.0%)	0.451
Malignancy	6 (8.6%)	9 (20.0%)	0.093	2 (8.7%)	4 (14.3%)	0.678
Immunosuppressive therapy	3 (4.3%)	5 (11.1%)	0.259	1 (4.3%)	2 (7.1%)	1.000
Obesity	20 (28.6%)	16 (35.6%)	0.537	5 (21.7%)	11 (39.3%)	0.232
Charlson comorbidity index	2.5 ± 3.2	4.8 ± 4.3	0.002	2.8 ± 3.0	4.1 ± 3.8	0.292
Infection data						
Prior surgery (within a month form infection onset)	34 (48.6%)	23 (51.1%)	0.850	10 (43.5%)	16 (57.1%)	0.404
Days at risk (from ICU admission to infection onset)	27.4 ± 26.5	21.5 ± 21.4	0.168	30.6 ± 35.2	21.6 ± 22.2	0.464
Isolate carrying *bla*_KPC_	59 (84.3%)	39 (86.7%)	0.794	18 (78.3%)	25 (89.3%)	0.442
Septic shock	23 (32.9%)	28 (62.2%)	0.002	-	-	-
Type of infection						
Primary BSI	34 (48.6%)	19 (42.2%)		12 (52.2%)	10 (35.7%)	
Catheter-related BSI	28 (40.0%)	7 (15.6%)		8 (34.8%)	4 (14.3%)	
Other^a^	8 (11.4%)	19 (42.2%)	<0.001^b^	3 (13.0%)	14 (50.0%)	0.007^b^
Antimicrobial treatment						
Monotherapy	1 (1.4%)	7 (15.6%)		0 (0.0%)	2 (7.1%)	
Combination of two antimicrobials	9 (12.9%)	13 (28.9%)		1 (4.3%)	7 (25.0%)	
Combination of three or more antimicrobials	60 (85.7%)	25 (55.6%)	<0.001	22 (95.7%)	19 (67.9%)	0.015
Carbapenem-including treatment	66 (94.3%)	40 (88.9%)	0.310	21 (91.3%)	26 (92.9%)	1.000
Colistin-including treatment	64 (91.4%)	38 (84.4%)	0.366	22 (95.7%)	25 (89.3%)	0.617
Aminoglycoside-including treatment	47 (67.1%)	20 (44.4%)	0.020	16 (69.6%)	14 (50.0%)	0.253
Tigecycline-including treatment	48 (68.6%)	24 (53.3%)	0.117	18 (78.3%)	20 (71.4%)	0.749
Corticosteroid administration during infection	37 (52.9%)	32 (71.1%)	0.055	13 (56.5%)	21 (75.0%)	0.234

Data are number (%) of patients or mean ± standard deviation. ^a^ Eleven intra-abdominal, eight ventilator-associated pneumonia, four nosocomial meningitis, three urinary tract, and one surgical site infection. ^b^ Comparison of other types of BSIs to primary and catheter-related BSIs

**Table 2:** Multivariate analyses of predictors of 14-day mortality in patients with bloodstream infection (BSI) during intensive care unit (ICU) hospitalization.

Characteristics	All Patients		Patients with Septic Shock	
	*p*	OR (95% CI)	*p*	OR (95% CI)
Septic shock	0.002	5.2 (1.8–15.0)		
Charlson comorbidity index	0.012	1.2 (1.0–1.3)		
BSI other than primary or catheter-related	0.001	6.4 (2.0–20.2)	0.008	7.4 (1.7–33.0)
Combination of at least three antimicrobials	<0.001	0.105 (0.032–0.344)	0.029	0.083 (0.009–0.774)

CI: confidence interval; OR: odds ratio.

**Table 3:** Types of antimicrobial treatment and the associated 30-day mortality.

Treatment Regimen	Survivors (*n* = 70)	Non-Survivors (*n* = 45)	Mortality (%)
One antimicrobial	1	7	87.5
Carbapenem	1	4	80.0
Colistin	0	2	100
Gentamicin	0	1	100
Two antimicrobials	9	13	59.1
Carbapenem + Aminoglycoside	2	1	33.3
Colistin + Tigecycline	0	1	100
Carbapenem + Tigecycline	2	1	33.3
Carbapenem + Colistin	5	10	66.7
Three antimicrobials	30	9	23.1
Colistin + Tigecycline + Aminoglycoside	2	0	0.0
Carbapenem + Tigecycline + Aminoglycoside	15	3	16.7
Colistin + Tigecycline + Carbapenem	10	5	33.3
Colistin + Carbapenem + Aminoglycoside	1	0	0.0
Colistin + Tigecycline + Ceftazidime/avibactam	1	0	0.0
Colistin + Tigecycline + Fosfomycin	1	1	50.0
Four or more antimicrobials	30	16	34.8
Carbapenem + Colistin + Tigecycline + Aminoglycoside	25	15	37.5
Carbapenem + Colistin + Tigecycline + Aminoglycoside + other antimicrobial	3	0	0.0
Double carbapenem + Colistin + Tigecycline + Aminoglycoside	2	0	0.0
Carbapenem + Colistin + Tigecycline + Fosfomycin	0	1	100

## Data Availability

The datasets generated during and/or analysed during the current study are available from the corresponding author on reasonable request.

## Supplementary Materials

The following are available online at https://www.mdpi.com/2079-6382/10/1/76/s1.
