# Supplementary figures and images for: Mortality of Pandrug-Resistant Klebsiella pneumoniae Bloodstream Infections in Critically Ill Patients: A Retrospective Cohort of 115 Episodes
Source: Antibiotics (Basel). 2021 Jan 15;10(1):76. doi: 10.3390/antibiotics10010076 (PMC7830393; doi:10.3390/antibiotics10010076)

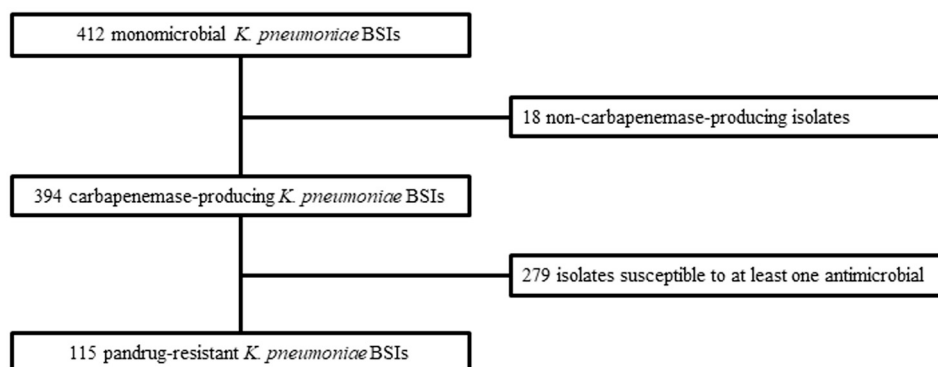

Supplement: Supplementary file 1 [file antibiotics-10-00076-s001.pdf]
